# Anthocyanin-rich fractions from red raspberries attenuate inflammation in both RAW264.7 macrophages and a mouse model of colitis

**DOI:** 10.1038/srep06234

**Published:** 2014-08-29

**Authors:** Li Li, Liyan Wang, Zhiqin Wu, Lijun Yao, Yonghou Wu, Lian Huang, Kan Liu, Xiang Zhou, Deming Gou

**Affiliations:** 1 College of Life Sciences, Shenzhen Key Laboratory of Microbial Genetic Engineering, Shenzhen University, Shenzhen 518060, China; 2 College of Life Sciences, Department of Marine Science and Bio-Pharm, Shenzhen Key Laboratory of Marine Bioresourse and Eco-environmental Science, Shenzhen 518060, China; 3 College of Animal Science and Technology, Northwest A&F University, Yangling, Shaanxi 712100, China; 4 College of Life Sciences, Shenzhen key laboratory of synthetic biology, Shenzhen University, Shenzhen 518060, China; 5 These authors contributed equally to this work.

## Abstract

Edible berries have a broad spectrum of biomedical functions, including improving immune responses and reducing risk for chronic diseases. In this study, the anti-inflammatory activities of crude extracts (CEs), anthocyanin-rich fractions (ARFs), and des-anthocyanin fractions (DAFs) from seven berries were evaluated based on their inhibitory effects on nitric oxide (NO) production in lipopolysaccharide (LPS)/IFN-γ-activated RAW264.7 macrophages. ARFs from red raspberries (RR-ARFs) exhibited the highest efficiency in suppressing NO synthesis. The anti-inflammatory properties were also demonstrated by reducing the expression levels of inducible nitric oxide synthase (iNOS), cyclooxygenase-2 (COX-2), interleukin-1 beta (IL-1β) and IL-6 in RAW264.7 cells. The luciferase reporter assay demonstrated that the activities of NF-κB and AP-1 signaling pathways were significantly suppressed by RR-ARFs. Further studies showed that RR-ARFs decreased the phosphorylation of IKK, IκBα, p65 and JNK and the nuclear translocation of p65 in LPS/IFN-γ-stimulated RAW264.7 cells. In a mouse colitis model, dextran sulfate sodium (DSS)-induced weight loss and histological damage were significantly ameliorated by RR-ARFs treatment. Taken together, our results indicate that RR-ARFs attenuate inflammation both in vitro and in vivo primarily by inhibiting the activation of NF-κB and MAPKs. The anti-inflammatory of RR-ARFs could be harnessed and applied in animal agriculture, drug and food industries.

Macrophages play important roles in the innate and adaptive immune responses by releasing various factors such as pro-inflammatory cytokines, oxygen, and nitrogen species. One critical releasing factor of nitric oxide (NO), an inorganic free-radical gaseous molecule, has been implicated in a variety of physiological and pathological processes, such as blood pressure regulation, vasodilatation induction, bone resorption, uterus dilation facilitation during pregnancy, penile erection production and maintenance, etc.^1^,^2^,^3^. A deficiency in NO production or availability is a hallmark of numerous disease conditions^1^,^2^,^3^. However, NO might cause unwanted negative effects due to its free radical chemistry. Excessive production of NO appears to associate with headaches, dizziness, low blood pressure and inflammatory diseases such as rheumatoid arthritis^4^. Inducible nitric oxide synthase (iNOS) is one of three key enzymes generating NO from the amino acid L-arginine^5^. iNOS mediates unspecific host defense mechanisms and plays critical roles in clearing bacterial, viral, fungal and parasitic infections^6^,^7^. It can be induced in many types of cells with suitable agents such as bacteria lipopolysaccharides (LPS) and cytokines including recombinant interferon-γ (IFN-γ)^8^. Therefore, inhibition of iNOS activation and NO production may be of therapeutic benefit against various types of inflammation^9^.

Epidemiologic studies have shown that the consumption of anthocyanin-rich fruits and vegetables is associated with lower risk of chronic disease development like arthritis, atherosclerosis, cardiovascular disease, cancer, and diabetes^10^,^11^,^12^,^13^,^14^. Berries contain high amounts of polyphenols such as anthocyanins, which are responsible for the red, purple and blue hues in fruits, and play important roles in plant physiology^15^. As a major source for dietary anthocyanin intake, berry research has traditionally focused on their antioxidant properties^16^,^17^,^18^,^19^; however, biologically, several studies suggested that their effects are not through antioxidant scavenging properties. Berry extracts and anthocyanin preparations have been shown to inhibit the development of of chronic disease by impacting specific steps in cell signaling pathways^20^,^21^,^22^. Recent pre-clinical data suggested important effects on inflammatory pathways^23^,^24^. Correspondingly, the effects of berries, including extracts and purified anthocyanins have been the subject of a number of human trials^25^.

The objective of current study was to evaluate the potential anti-inflammatory activities, seven different berry crude extracts (CEs), anthocyanin-rich fractions (ARFs) and des-anthocyanin fractions (DAFs) by quantifying their inhibitory effects on NO production in LPS/IFN-γ activated RAW264.7 macrophages. Our results showed that ARFs from red raspberries (RR-ARFs) have the highest inhibitory effect on NO production in a dose-dependent manner. For the first time, we demonstrated the anti-inflammatory effect of RR-ARFs *in vitro* and *in vivo* and revealed the involvement of NF-κB and MAPKs.

## Results

### The contents of anthocyanin-rich fractions (ARFs) from different berry extracts

Solid-phase extraction (SPE) using the absorbent Amberlite XAD-7 was applied to extract ARFs from seven berry species. The amounts of ARFs in different berry extracts (500 mg/each) were varied from 58.2 mg for Kiown to 93.1 mg for black raspberry crude extract (Table 1). Overall, black raspberry, mulberry and red raspberry had higher ARFs contents than blackberries. Of the four types of blackberries, the ARFs from cultivar of Triple Crown (77.0 mg) were higher than those of Shawnee (58.8 mg), Chester (59.2 mg), and Kiown (58.2 mg).

### Evaluation of the cytotoxic effects of different berry extracts on RAW264.7 Cells

**Figure 1:**
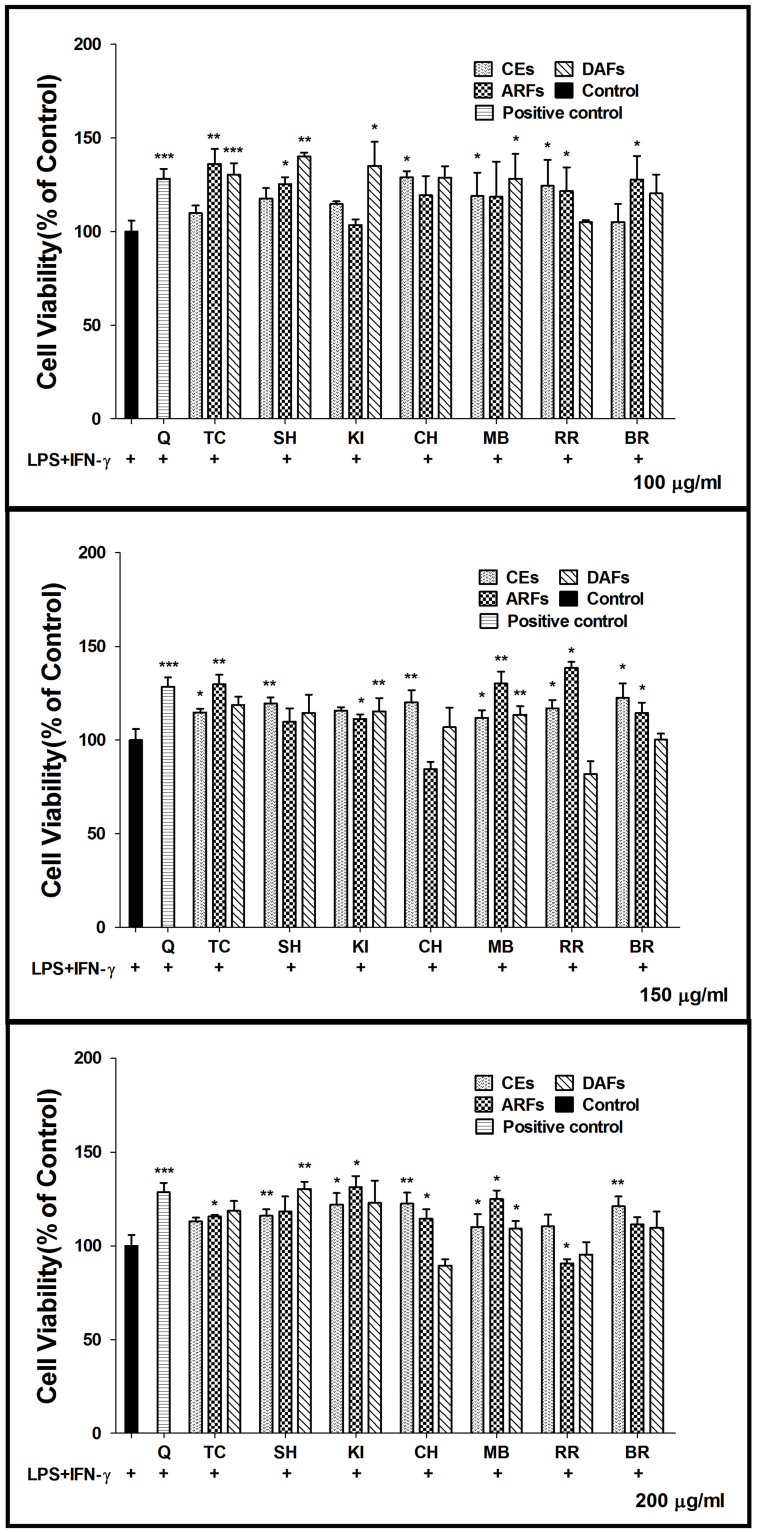
Effects of the berry extracts on cell viability. RAW264.7 cells were cultured in the presence or absence of berry extracts at three different concentrations (100, 150 and 200 μg/ml), which referred to the concentrations of dried mixture of berry extracts, and the cell viability was determined by the MTS assay. Each value represents mean ± SD from six independent experiments. Quercetin (Q), Triple Crown (TC), Shawnee (SH), Kiown (KI), Chester (CH), Mulberry (MB), Red raspberry (RR), BR (Black Raspberry).

The cytotoxicity of berry extracts was evaluated using the MTS assay. RAW264.7 cells were incubated with dried mixture of berry extracts (CEs, ARFs and DAFs) at three different concentrations (100, 150 and 200 μg/ml). The data from MTS assay showed no significant changes in cell viability, indicating that all berry extracts (CEs, ARFs and DAFs) were not cytotoxic at dosage up to 200 μg/ml (Fig. 1).

### Effect on NO production

**Figure 2:**
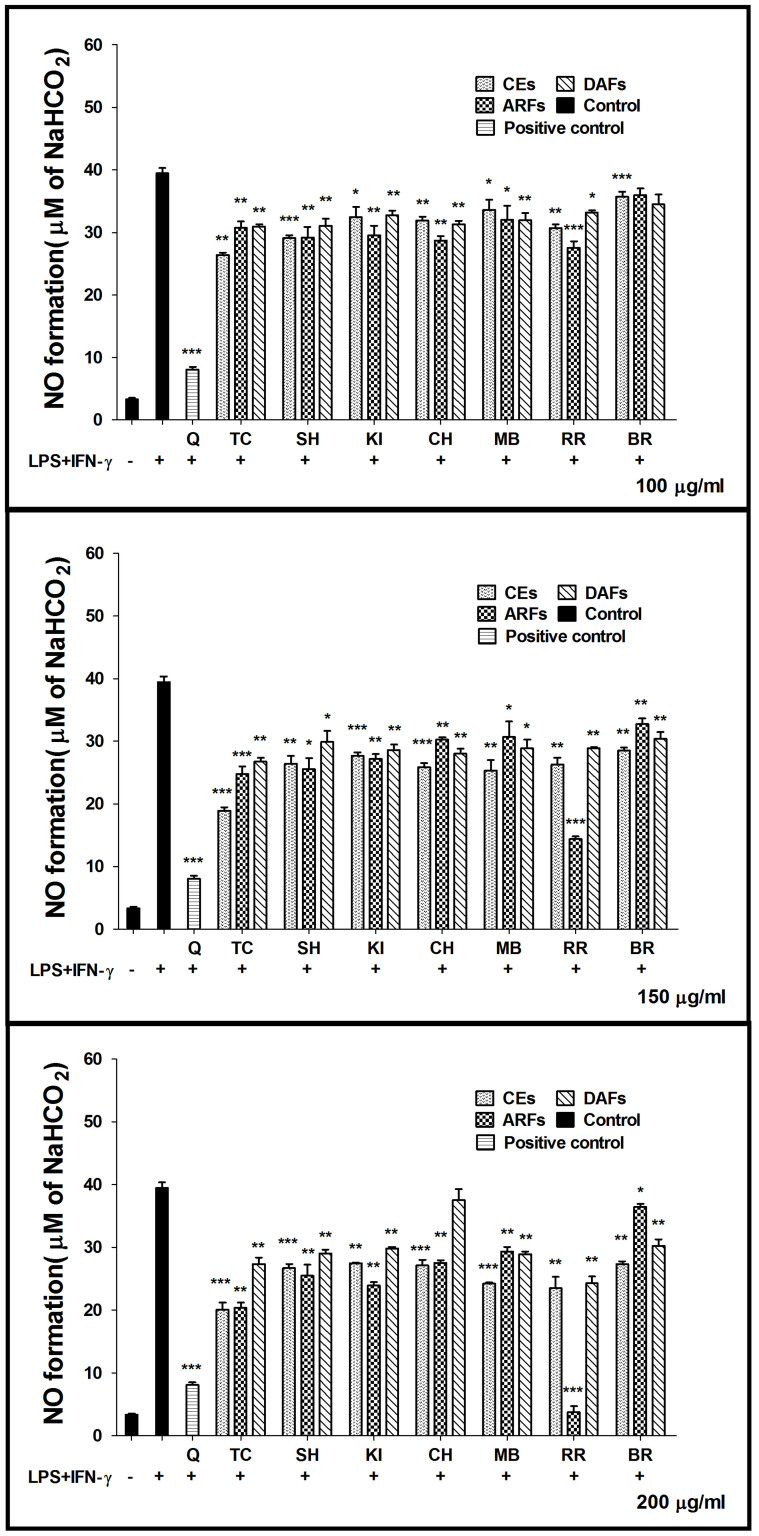
Effects of berry extracts on NO synthesis in LPS/IFN-γ-activated RAW264.7 cells. RAW264.7 cells were treated with LPS (1.5 μg/ml)/IFN-γ (10 ng/ml) alone or together with each extract at concentrations indicated. After 24 h incubation, the culture media were collected for NO measurement using the Griess assay. The data were expressed as the means ± SD from six individual experiments. * *P* < 0.05,** *P* < 0.01,*** *P* < 0.001 compared to LPS/IFN-γ control.

Flavonoids and polyphenolic compounds have been shown to inhibit NO production^17^. To investigate if the berry extracts have anti-inflammatory properties, NO production was measured in the presence or absence of different berry extracts. When LPS/IFN-γ were administered to RAW264.7 macrophages, the NO production increased dramatically from the basal level of ~3 to 40 μM after 24 h incubation (Fig. 2), indicating the successful activation of cells by LPS/IFN-γ treatments. Dose-dependent inhibition of NO production was observed when cells were co-treated with various concentrations of different berry extracts (Fig. 2). Most berry fractions moderately inhibited NO production (about 20.70%–25.47%) at non-toxic concentrations (150, 200 μg/ml). Most of the ARFs had the same range of inhibitory effects as CEs and DAFs. However surprisingly, ARFs from RR (RR-ARFs) showed a robust inhibition of NO synthesis by 69.34% and 98.84% at 150 and 200 μg/ml, respectively (Fig. 2), which were much higher than those of other berry extracts. As the most abundant flavonoids in the human diet, Quercetin was used as a positive control in this study. We observed that a treatment of Quercetin (Sigma) at 12.5 μg/ml caused 79.59% inhibition of NO production, which is lower than the RR-ARFs treatment at a higher concentration (200 μg/ml), further indicating that RR-ARFs have notable anti-inflammatory potency.

### RR-ARFs inhibit iNOS and COX-2 expression

**Figure 3:**
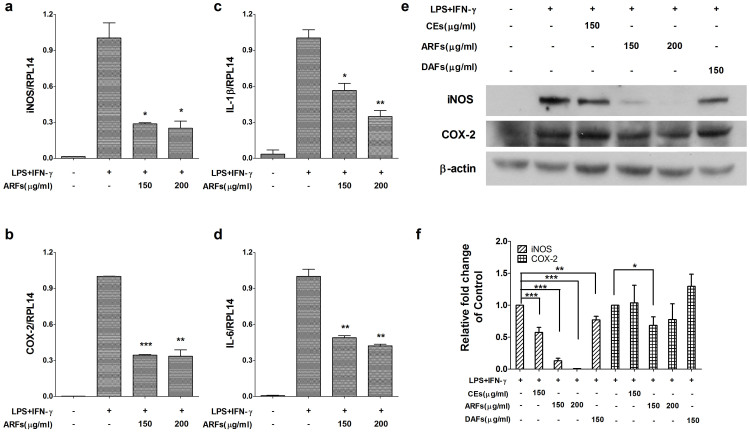
RR-ARFs inhibit LPS/IFN-γ-mediated iNOS, COX-2, IL-1β and IL-6 expressions in RAW264.7 cells. Cells were incubated with different extracts of RR and LPS/IFN-γ for 16 h respectively at concentrations indicated. The expressions of mRNA for iNOS (a), COX-2 (b), IL-1β (c) and IL-6 (d) were analyzed by real-time PCR normalized to RPL14 mRNA. iNOS (130 kDa) and COX-2 (73 kDa) protein levels (e) were examined using Western blot. β-actin was used as an internal loading control. The full-length blots with anti-iNOS, anti-COX-2 and anti-β-actin antibodies were presented in supplementary Figure S1. All gels have been run simultaneously under the same experimental conditions. Cell culture experiments were performed at least three times. The relative fold changes of iNOS and COX-2 protein were compared with LPS/IFN-γ control (f). Values are expressed as the means ± SD of at least three independent experiments. * *P* < 0.05, ** *P* < 0.01, *** *P* < 0.001 compared to LPS/IFN-γ control.

The iNOS gene is known to be the primary regulator of NO production in macrophages and the COX-2 gene is often increased under inflammatory and malignant conditions^5^,^26^. Therefore, the effects of RR-ARFs on the expression of iNOS and COX-2 were measured by real-time RT-PCR and Western blot analyses. As shown in Fig. 3, the expression level of iNOS and COX-2 in RAW264.7 cells were significantly elevated upon LPS/IFN-γ treatment. Notably, the expression of iNOS at both mRNA and protein levels as well as the COX-2 mRNA expression were significantly reduced by the treatment of RR-ARFs in a concentration-dependent manner (Fig. 3a, 3b, 3e and 3f). COX-2 protein level was also suppressed by the RR-ARFs treatment (Fig. 3e and 3f). RR-CEs and RR-DAFs had little effects on activation of iNOS and COX-2 (Fig. 3e).

### RR-ARFs inhibit IL-1β and IL-6 mRNA expression

We also investigated whether RR-ARFs inhibit LPS/IFN-γ-induced pro-inflammatory cytokines IL-1β and IL-6 mRNA expression. As shown in Fig. 3c and 3d, RR-ARFs significantly decreased the mRNA levels of IL-1β and IL-6 in a concentration-dependent manner.

### RR-ARFs inhibit the activities of NF-κB and AP-1 pathway reporters

**Figure 4:**
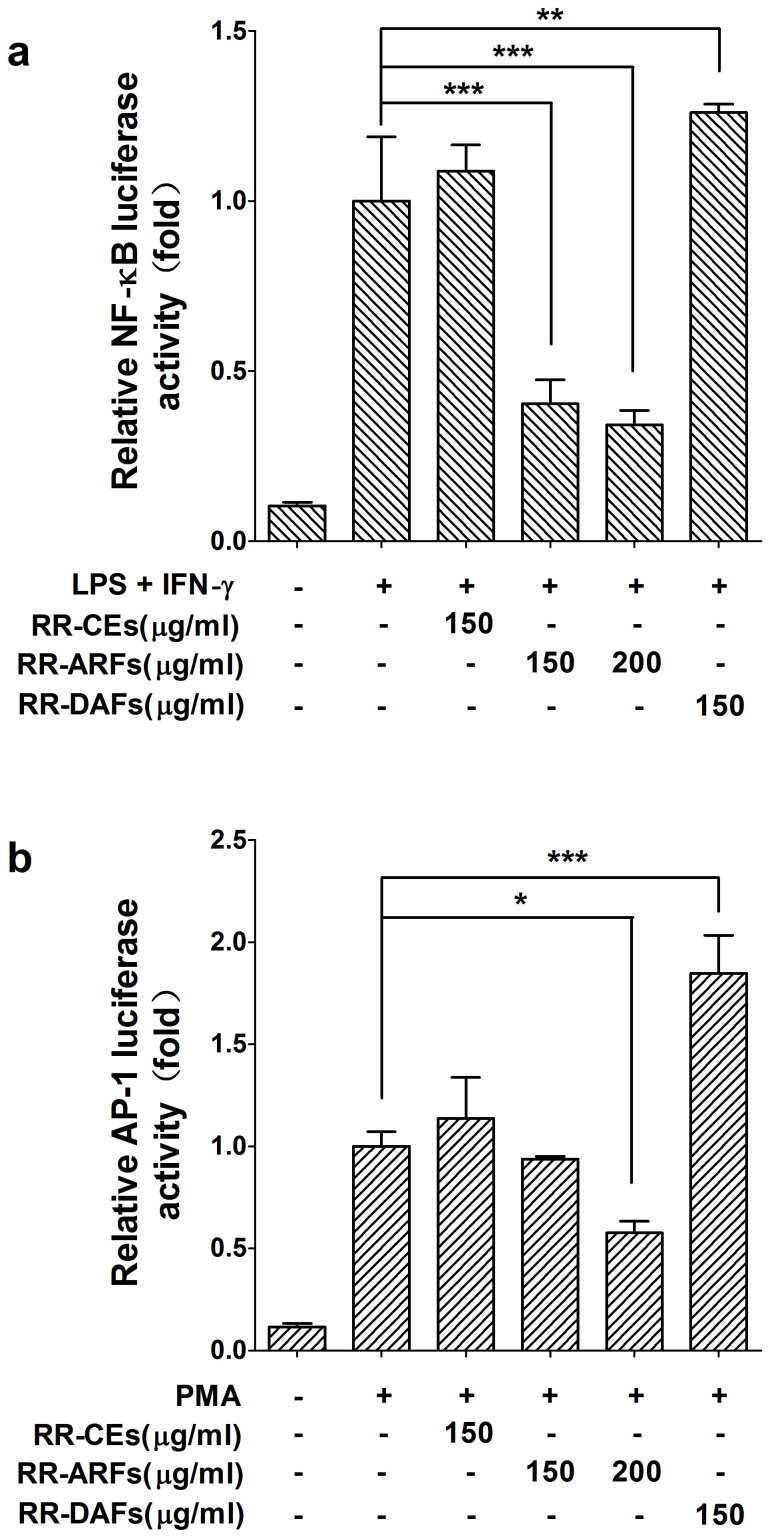
NF-κB and AP-1 pathway luciferase reporter assays. RAW264.7 cells were transiently co-transfected with pNFκB-Luc (a) or pAP1-Luc (b) and pRL-TK using Lipofectamine™ 2000 Transfection Reagent. Cells were then treated with different extracts from RR and LPS/IFN-γ or PMA. After 24 h culture, cells were collected for dual-luciferase activity assays. Each value represents the mean ± SD of three independent experiments, and * P < 0.05, ** P < 0.01, *** P < 0.001 compared to LPS/IFN-γ control.

Since LPS/IFN-γ-induced iNOS and COX-2 expression are transcriptionally dependent on the transcription factors of NF-κB and AP-1^27^, we investigated the impact of RR-ARFs on these two important pathways using pathway luciferase reporter assays. As shown in Fig. 4a and 4b, both NF-κB and AP-1 transcriptional activities were inhibited by the treatments of RR-ARFs (150, 200 μg/ml) when compared to each corresponding control. However, the activities were not decreased by the treatment of RR-CEs (150 μg/ml) and RR-DAFs (150 μg/ml). These results suggested that RR-ARFs inhibit the secretion of NO via suppressing NF-κB and AP-1 signaling pathways in RAW264.7 cells.

### RR-ARFs prevent NF-κB subunit p65 nuclear translocation

**Figure 5:**
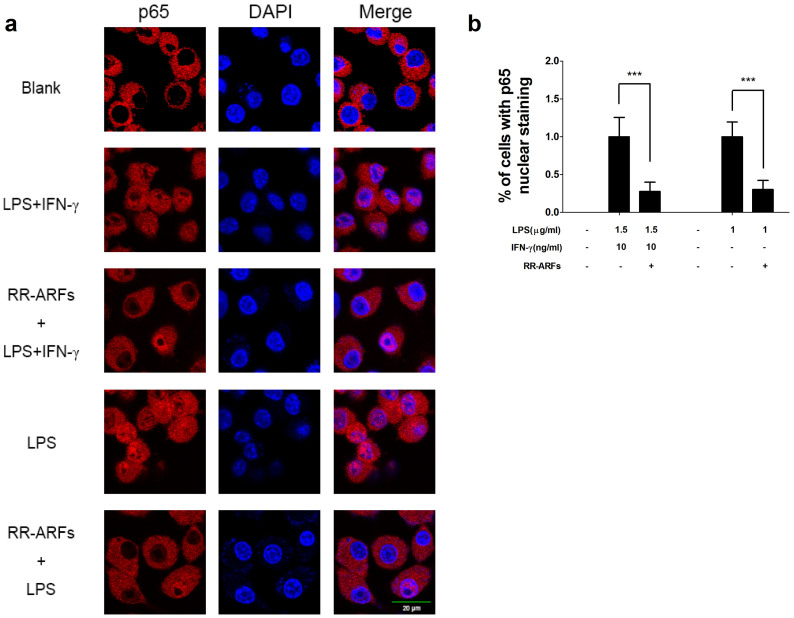
RR-ARFs inhibit LPS/IFN-γ-induced nuclear translocation of NF-κB (p65). RAW264.7 cells were pretreated with or without RR-ARFs (200 μg/ml) for 12 h and then exposed to LPS/IFN-γ or 1 μg/ml LPS only for 1 h. Then cells were fixed, permeabilized and immunofluorescent staining performed for p65. Nuclei were stained with DAPI (a). The relative percentage of p65 translocation compared to LPS/IFN-γ control was quantified based on three independent experiments, and *** P < 0.001 (b).

Since the nuclear translocation of the NF-κB transcriptional subunit p65 is a critical step for the activation of NF-κB signaling pathway, we investigated if RR-ARFs affect the subcellular compartmentalization of p65. RAW264.7 cells were pretreated with RR-ARFs (200 μg/ml) for 12 h and then stimulated with LPS/IFN-γ or LPS for another 1 h. Cellular localization of p65 were determined by immunofluorescence with confocal microscopic analysis. As expected, p65 was mainly translocated to the nucleus in LPS/IFN-γ or LPS stimulated RAW264.7 cells. However, pre-treatment of RR-ARFs significantly reduced the p65 nuclear accumulation when compared to that in the control cells with LPS/IFN-γ or LPS only (Fig. 5). These results demonstrated that RR-ARFs prevent p65 nuclear translocation.

### RR-ARFs inhibit LPS/IFN-γ-induced activation of IKK and IκBα

**Figure 6:**
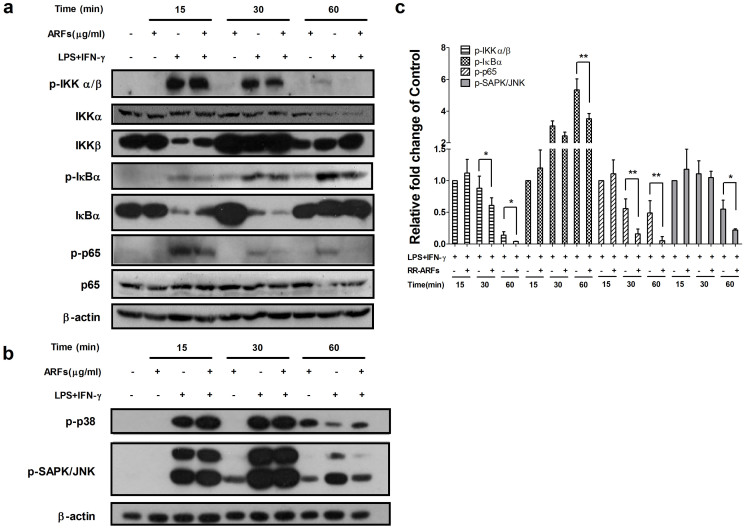
Effects of RR-ARFs on IKK and MAPK signaling in LPS/IFN-γ-stimulated RAW264.7 cells. Cells were stimulated with LPS/IFN-γ in the absence or presence of RR-ARFs (200 μg/ml) for the indicated time. The protein levels of phospho-IKKα/β (85–87 kDa), IKKα (85 kDa), IKKβ (87 kDa), phospho-IκBα (40 kDa), IκBα (39 kDa), phospho-p65 (65 kDa), p65 (65 kDa), phospho-p38 (43 kDa) and phospho-JNK (46 and 54 kDa) were detected by Western blot analyses using their respective antibodies (a and b). β-actin was used as an internal loading control. The full-length blots with those antibodies were presented in supplementary Figure S2. All gels have been run simultaneously under the same experimental conditions. Cell culture experiments were performed at least three times. Western blot data was quantitated to better view the difference between different treatment groups. Data represent means ± SEM; * P < 0.05, ** P < 0.01, *** P < 0.001 (c).

To identify the up-stream targets of ARF-mediated NF-κB blockade, we investigated the status of IKK and IκBα phosphorylation/degradation. RAW264.7 cells were stimulated with LPS/IFN-γ for 0, 15, 30 and 60 min in the absence or presence of RR-ARFs (200 μg/ml). As shown in Fig. 6a and 6c, the phosphorylation of IKK was rapidly induced by LPS/IFN-γ at 15 min stimulation, then decreased by 12% and 86% at 30 and 60 min, respectively. However, RR-ARFs negatively regulated the phosphorylation of IKK, where it had little effect on the total protein of IKKα. It is very notable observation that the phosphorylation of IκBα was significantly inhibited by RR-ARFs at 30 and 60 min LPS/IFN-γ stimulation. The phosphorylated p65 at 30 and 60 min stimulation were also suppressed by RR-ARFs treatments. These results indicated that RR-ARFs inhibit NF-κB activation via blocking the phosphorylation of IκBα and its upstream kinase IKK.

### RR-ARFs inhibit the LPS/IFN-γ-induced activation of JNK

The key transcription factors involved in the regulation of iNOS expression are NF-κB and AP-1^28^,^29^. AP-1 is composed of proteins belonging to the Jun and Fos families. It is well known that MAPK signaling pathways play an essential role in regulating AP-1 activity by increasing its transcription and/or phosphorylation. Since we have observed that RR-ARFs negatively regulate AP-1 activity, we investigated whether the inhibition of NO production by RR-ARFs is also associated with MAPK signaling pathways. As expected, LPS/IFN-γ rapidly induced the phosphorylation of p38 and JNK. Interestingly, RR-ARFs inhibited the phosphorylation of JNK after 60 min stimulation, however, the phosphorylation of p38 did not change significantly (Fig. 6b and 6c). These findings suggested the ability of RR-ARFs to suppress JNK activation, which is consistent with the inhibition of AP-1 transcriptional activation in LPS/IFN-γ -stimulated RAW264.7 cells.

### RR-ARFs alleviate DSS-induced colitis

**Figure 7:**
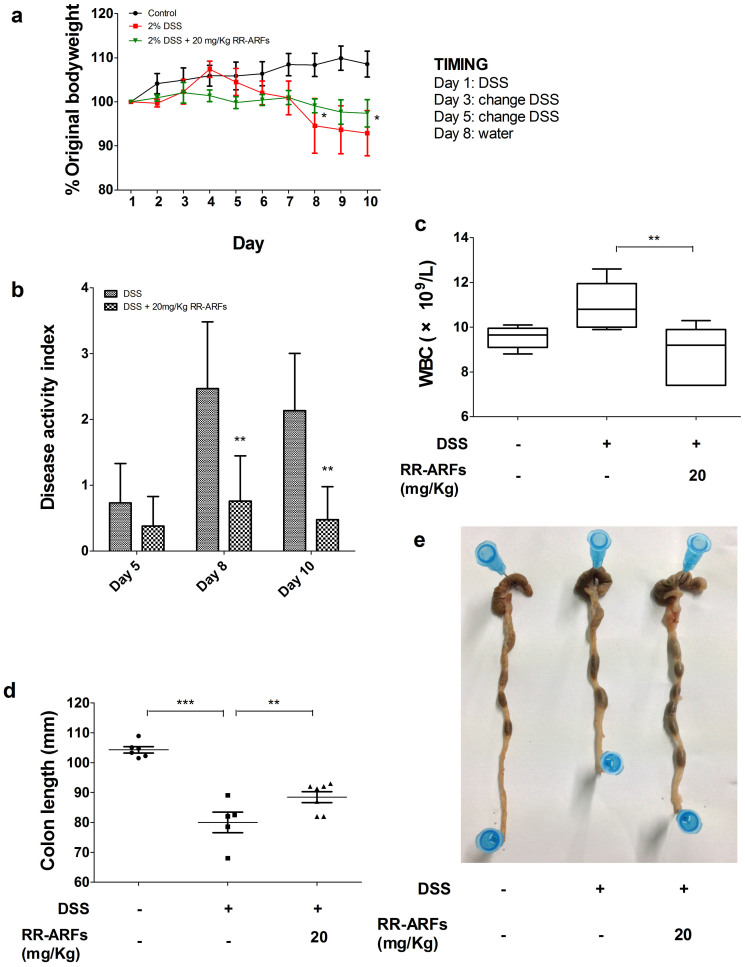
RR-ARFs alleviate DSS-induced pathology. BALB/c mice were injected with RR-ARFs (20 mg/kg) 1 day prior to exposure to 2% DSS in drinking water. Daily weights were measured (n = 6–8/group) and plotted as percentage body weight change from initial weight, expressed as group percentage mean ± SEM (a). Disease activity index (b). Hematological parameters was tested using MINDRAY BC-3000 PLUS Automatic Hematology Analyzer (c). Analysis of colon length from experimental mouse groups (d and e). Representative of two independent experiments. * P < 0.05, ** P < 0.01 compared to DSS-only control.

**Figure 8:**
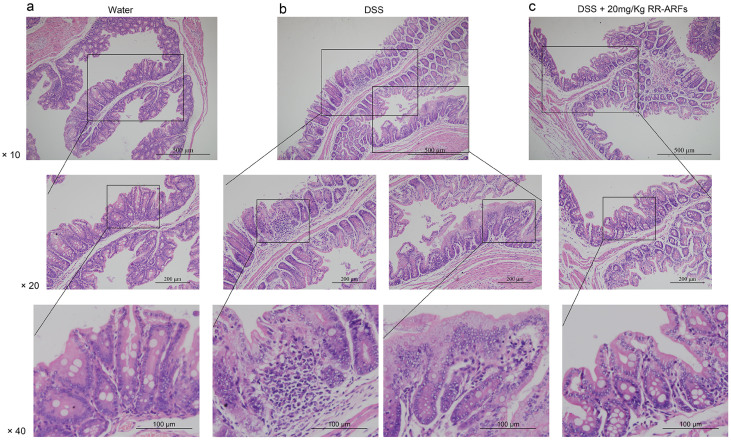
Effects of RR-ARFs on colorectal histology in mice with DSS-induced colitis. Histological changes were determined by hematoxylin and eosin staining. Water group showing normal histology of mice colon (a). Mucosal injury produced after DSS administration with distortion of crypts (b). DSS + 20 mg/kg RR-ARFs samples showing a reduction in the morphological alteration associated to DSS treatment (c).

To test the physiological relevance of RR-ARF-mediated suppression of inflammatory reactions *in vivo*, a DSS model of acute intestinal inflammation was used. The administration of DSS induced experimental colitis, characterized by weight loss, shortened colon, and the production of bloody stools as indicated by the disease activity index (DAI) score (Fig. 7b). In this model, RR-ARFs significantly reduced body weight loss and colon shortening (Fig. 7a, 7d and 7e). DSS treatment caused approximately 8% weight-loss in control mice, whereas mice treated with RR-ARFs (20 mg/kg) were significantly protected from weight loss on d8 and d10 (Fig. 7a; *P* < 0.05). Moreover, we measured the hematological parameters and found that RR-ARFs significantly reduced WBC counts (Fig. 7c; *P* < 0.01). While control mice had morphologically normal colons with no inflammation (Fig. 8a), an obvious distortion of crypts was observed in mice after DSS administration (Fig. 8b). According to a five-point scoring system for inflammation evaluation (Table 2), RR-ARFs treatment showed lower level of inflammation with scattered infiltrating mononuclear cells in DSS-treated mice (Table 3) and a reduction in the morphological alteration (Fig. 8c). Taken together, these findings showed that RR-ARFs reduced DSS-induced acute colitis.

## Discussion

Recent studies compared the anti-inflammatory effects of anthocyanins from the blueberry, blackberry, and blackcurrant by measuring the production of proinflammatory cytokines and found similar anti-inflammatory activities despite their anthocyanin composition and total antioxidant capacity values are markedly different^24^. In this study, we focused on seven popular consumed berries cultured in China and evaluated the anti-inflammatory activities of CEs, ARFs and DAFs by measuring their effects on the production of NO in LPS/IFN-γ-induced RAW264.7 cells. Almost all of the extracts (ARFs, CEs, and DAFs) from different berries displayed similar ranges of NO inhibition, which is consistent with previous studies^24^. Nevertheless, ARFs from red raspberries exhibited the strongest NO inhibitory activity, with a nearly complete inhibition at 200 μg/ml, which was not caused by cytotoxicity. To our knowledge, there is no published report on the NO inhibition by RR-ARFs. Therefore, we tried to identify its role and underlying molecular mechanism in preventing NO production.

LPS stimulation of macrophages (RAW264.7) is a widely used *in vitro* model for evaluating the potency of anti-inflammatory drugs and exploring their mechanisms of action^30^. LPS can activate several extracellular signaling pathways, including NF-κB and MAPKs^28^,^31^. The transcription factor of NF-κB is primarily composed of the proteins p50 and p65, and it is inactive when present in the cytosol bound to the inhibitory protein of IκB. Upon exposure to proinflammatory stimuli, such as bacterial LPS, TNF-α, or IL-1, the IκB are rapidly phosphorylated by IκB kinase (IKK) and triggers its own proteolytic degradation, releasing NF-κB, which then translocates to the nucleus and regulates the expression of numerous genes related to growth, development, apoptosis, inflammation, and oncogenesis^32^. To show how RR-ARFs treatment reduced the level of NO in LPS/IFN-γ-induced RAW264.7 cells, we first examined the expression of iNOS and COX-2, two major inflammatory mediators. Both mRNA and protein levels of iNOS and COX-2 were markedly diminished by RR-ARFs in a dose-dependent manner. Moreover, two proinflammatory cytokines, IL-1β and IL-6 were also decreased by RR-ARFS as part of the inflammatory response. Since the transcription factors of NF-κB and AP-1 are two major regulators in controlling iNOS and COX-2 expression, we then performed pathway luciferase reporter assays in LPS/IFN-γ-induced (for NF-κB pathway) or PMA-induced (for AP-1 pathway) RAW264.7 cells with or without RR-ARFs treatments. RR-CEs and RR-DAFs were also included for comparison purposes. Our data showed that only RR-ARFs could obviously inhibit NF-κB pathway reporter activities, which can be further enhanced by the high dosage of RR-ARFs. The activity of AP-1 pathway reporter was also diminished by RR-ARFs at higher concentration (200 μg/ml). RR-DAFs treatment increased the AP-1 pathway activity at unknown reason. These data indicated that RR-ARFs induced anti-inflammatory property is mediated through NF-κB and AP-1 signaling pathways.

Next, we examined the essential upstream kinases in controlling NF-κB p65 subunit activation. We found that RR-ARFs blocked the LPS/IFN-γ-induced phosphorylation of IκBα and its upstream kinase IKK, therefore, resulting in the decrease of phosphorylated/activated p65 and its translocation from cytosol to nucleus. As MAPKs are also important pathways involved in LPS-induced iNOS expression in mcrophages, we also investigated the effects of RR-ARFs on LPS/IFN-γ-induced activation of the MAPK pathways in RAW264.7 cells. We found that RR-ARFs inhibit the phosphorylation of JNK but not p38. Thus, we conclude that RR-ARFs-induced anti-inflammatory response is primarily mediated through the suppression of IKK, IκBα and JNK activation.

To determine whether RR-ARFs can protect against and treat colonic inflammation *in vivo*, we used a mouse model of DSS-induced acute colitis. The DSS colitis model showed RR-ARFs led to a significant amelioration of several disease activity parameters. This effect of treatment with RR-ARFs on the clinical signs of the disease included decreases in body weight loss and colon shortening as well as a reduction in WBC counts in the BALB/c mice suffering from this acute inflammatory condition.

**Figure 9:**
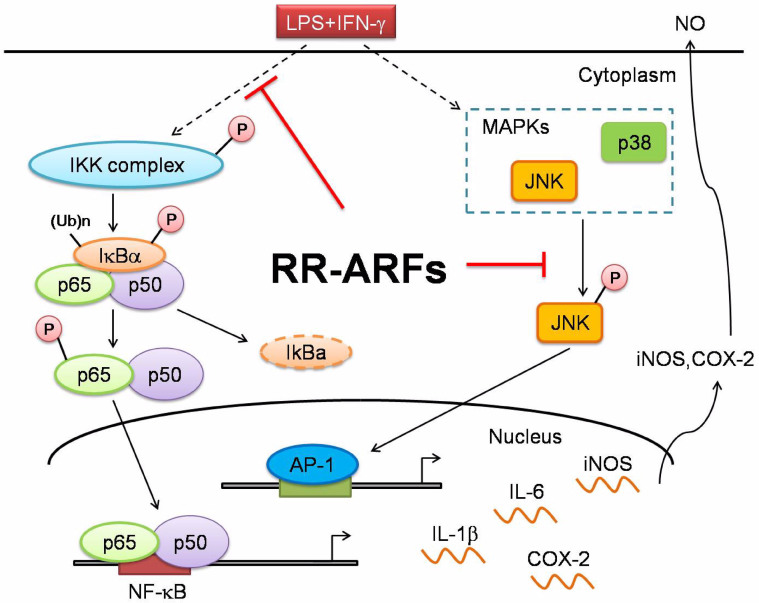
Schematic model showing role of RR-ARFs in inflammatory signaling pathways. RR-ARFs attenuated LPS/IFN-γ-induced inflammatory responses through inhibition of NF-κB, MAPK/JNK activities, respectively, in LPS/IFN-γ-stimulated RAW264.7 cells.

In conclusion, RR-ARFs attenuate LPS/IFN-γ-induced inflammatory responses in RAW264.7 cells by blocking activation of NF-κB and MAPK/JNK as shown in Fig. 9. The administration of RR-ARFs significantly inhibited p65 phosphorylation and its nuclear translocation as well as the activation of IKK, IκBα and JNK, thereby suppressing the expression of proinflammatory genes such as iNOS, COX-2, IL-1β, and IL-6. Moreover, RR-ARFs reduced the severity of colitis, as assessed based on histology in an acute mouse colitis model. These findings suggested that RR-ARFs could be potentially developed as a potentially anti-inflammatory agent for clinical and agriculture applications. According to a recent study by Johnson et al^33^, the anthocyanin biosynthesis could be influenced by environmental factors such as temperature, production site, and geography. Different varieties of the same species also have varying amounts of anthocyanins. In our future work, we will identify potential variations of RR-ARFs from different sources and test their anti-inflammatory activities in murine and human cells. Our goal is to develop a dietary supplement from efficacious red raspberries that can contribute to human health.

## Methods

### Plant materials, preparation and fractionation of berry extracts

Mulberries (*Morus alba L*), red raspberries (*RubusidaeusL*) and black raspberries (*Rubusoccidentalis*) were grown in Liao Ning, China. Four types of blackberries (*Rubusfruticosus*) (different cultivar known as Triple Crown, Shawnee, Kiown, Chester) were obtained from local farmers in Henan province, China. Immediately after harvest, all fruits were washed and frozen at −80°C. The edible parts of the berries were applied for the preparation of crude extracts (CEs), anthocyanin-rich fractions (ARFs) and des-anthocyanin fractions (DAFs) as previously described^15^. Frozen berries were separately blended with methanol containing 0.1% HCl, filtered to separate purple pigment from pulp through a cotton filter for three times, and centrifuged. The supernatant was concentrated in a rotary evaporator (EYELA) for the preparation of the berry CEs. The same amount of supernatant before concentration was loaded into an Amberlite XAD-7 (Sigma) column, then rinsed with distilled water. Unbounded materials of mainly free sugars and organic acids were collected as DAFs. After extensive washes, the anthocyanin and proanthocyanidin containing ARFs were eluted with 100% methanol. CEs and DAFs were lyophilized to yield solid products, and ARFs were evaporated to dry in rotary evaporator. All berries were purchased two times from a same supplier. Each sample was extracted at least 3 times for the evaluation of their potency.

### Cell culture and treatment

Mouse RAW264.7 macrophage cells purchased from American Type Culture Collection (ATCC) were cultured in RPMI-1640 medium with 10% fetal bovine serum (Hyclone). It is essential to subculture the cells before reaching 90% confluence. Cells were plated in 96-well plate at a density of 1.0 × 10^5^ cells/well and grown for 2 h to allow cells to attach to plate. Berry extracts tested were dissolved in DMSO, and then made 2-fold serial dilutions in RPMI-1640. The final concentrations of dried extracts in the culture medium were 100, 150, 200 μg/ml. Quercetin (Sigma) was used as the positive control and the final concentration was 12.5 μg/ml. The extracts were added to the culture simultaneously with both *Escherichia coil* LPS (1.5 μg/ml, Sigma) and recombinant mouse IFN-γ (10 ng/ml, Peprotech). Cell viability and nitrite concentration in culture medium were determined 24 h after incubation. Control cells were grown under identical conditions without the test extracts.

### Cell viability assay

Cell viability was determined using the CellTiter 96 Aqueous One Solution Proliferation Assay Kit (Promega). The treated cells were incubated for 24 h. Then growth medium was replaced by a solution of 100 μl of fresh growth medium and 20 μl of MTS/PMS [(3-(4,5-dimethylthiazol-2-yl)-5-(3-carboxymethoxyphenyl) -2-(4-sulfophenyl)-2H-tetrazolium, inert salt, and an electron coupling reagent phenazinemethosulfate (PMS)] in each well. The plate was incubated for another 2 h at 37°C, and the absorbance was measured at 490 nm. The percentage of cell viability relative to DMSO (solvent control) was calculated.

### Nitrite measurement

NO levels in the culture media were determined by Griess Reagent System according to manufacturer's instructions (Promega). Briefly, 50 μl of cell culture supernatant was transfered into a 96-well plate and mixed with 100 μl of Griess reagent (equal volumes of 1% w/v sulfanilamide in 5% v/v phosphoric acid and 0.1% w/v naphthylethylenediamine-HCl). Then, the plate was incubated for 5–10 min at room temperature, and absorbance was measured at 550 nm using a microplate reader. A standard sodium nitrite curve was used to calculate the amount of NO (y = 0.0067 x + 0.0449, R^2^ = 0.9999).

### Luciferase reporter assay

The pathway reporter of pNFκB-Luc and pAP-1-Luc plasmids, which containing NF-κB or AP-1 binding sites in the promoter region, were purchased from Clontech (Mountain View). Overnight cultured RAW264.7 cells in 96-well plates were transfected with 100 ng of pNFκB-Luc or pAP-1-Luc reporter vector and 5 ng of phRL-TK Renilla luciferase reporter (Promega) using Lipofectamine™ 2000 transfection reagent (Invitrogen). 16 h after transfection, cells were treated with various concentrations of berry extracts and LPS/IFN-γ (for NF-κB reporter) or PMA (for AP-1 reporter), simultaneously. After another 24 h, the cells were lysed for Firefly/Renilla luciferase activity assay using a dual-luciferase assay kit (Promega). The relative luciferase activities were normalized by LPS/IFN-γ or PMA control.

### NF-κB immunofluorescence

RAW264.7 cells overnight grown on chamber slides were pretreated with RR-ARFs for 12 h, then stimulated with LPS or LPS/IFN-γ for 1 h. After washing with phosphate-buffered saline (PBS, pH 7.4), cells were fixed immediately in 4% formaldehyde for 20 min. Cells were permeabilized with 0.5% Triton X-100 for 20 min at room temperature. The slides were then incubated with a primary antibody against NFκB p65 (1:100 dilution) at 4°C overnight. Slides were washed and incubated with Cy3-conjugated anti-rabbit IgG secondary antibodies at 1:500 dilution (Jackson ImmunoResearch) at room temperature for another 1 h. Finally, the slides were washed and stained with 4′, 6′-diamidino-2-phenylindole (DAPI) for nuclei staining. The translocation of p65 into the nucleus was determined by confocal microscopy (Olympus).

### Quantification RT-PCR

To measure mRNA expression levels, total RNAs were extracted with RNAiso Plus (TaKaRa Biotechnology) and quantified using the NanoDrop 2000c Spectrophotometer (Thermo Fisher Scientific). One μg of DNase-treated total RNAs were reverse-transcribed with oligo(dT)_18_ (Takara Biotechnology) plus random hexamer primers (Takara Biotechnology) using M-MLV Reverse Transcriptase (Takara Biotechnology). The transcription levels of iNOS, COX-2, IL-6, IL-1β and house keeping control (RPL14) were quantified on Step-One plus real-time PCR System (Applied Biosystems) using SYBR green-I Master PCR Mix with specific real-time PCR primer sets (Forward/reverse primer sequences, for iNOS: 5′- CAG CTC AAG AGC CAG AAA CG -3′/5′-TTA CTC AGT GCC AGA AGC TG-3′; for COX-2, 5′-CCA CTT CAA GGG AGT CTG GA-3′/5′-AGT CAT CTG CTA CGG GAG GA-3′; for IL-6, 5′-CCA ATT TCC AAT GCT CTC CT-3′/5′-ACC ACA GTG AGG AAT GTC CA-3′; for IL-1β, 5′-CTT TGA AGA AGA GCC CAT CC-3′/5′-TTT GTC GTT GCT TGG TTC TC-3′, for RPL14, 5′-GGC TTT AGT GGA TGG ACC CT-3′/5′- GGC TTT AGT GGA TGG ACC CT-3′). All reactions were performed in triplicate. The mRNA expression level of each gene was normalized to RPL14 and calculated using the 2^−ΔΔCT^ method.

### Western blot analysis

Cells were collected and dissolved in RIPA mammalian protein extraction lysis buffer (50 mM Tris-HCl [pH 7.5], 150 mM NaCl, 1% NP-40, 0.25% sodium deoxycholate, and 1 mM EDTA) supplemented with protease inhibitor cocktail (Roche). Same amount of protein samples (whole-cell extracts, 30 μg/lane) were then separated by 10% SDS–polyacrylamide gel (PAGE) and transferred to polyvinylidene fluoride (PVDF) or nitrocellulose membranes. The membranes were blocked with 5% (w/v) non-fat milk, washed in Tris-buffered saline Tween-20 (TBST) solution, and then probed with primary antibodies against iNOS, COX-2, phospho-IKKα/β, IKKα, IKKβ, phospho-IκBα, IκBα, p65, phospho-p65, phospho-p38, phospho-JNK (Cell Signaling Technology), and β-actin (Proteintech) overnight at 4°C. After rinsing, membranes were incubated with horseradish peroxidase conjugated secondary antibody (Bio-Rad) at dilutions of 1:10,000 for 1 h at room temperature. Immunoreactive bands were detected by exposed to X-ray film and their densities were quantified using Carestream Molecular Imaging Software. Western blot data was quantitated to better view the difference between different treatment groups.

### DSS-induced acute colitis

BALB/c mice of 6- to 8-weeks of age were maintained in standard housing cages in a specific pathogen free (SPF) environment and allowed to have free access to feed and water. RR-ARFs and saline solutions were prepared daily and filter-sterilized prior to intraperitoneal (i.p) injection. Mice were injected with 20 mg/kg RR-ARFs for 10 days till the end of the experiment, with the first administration occurring 1 day prior to exposure to 2% DSS in drinking water. Mice were monitored daily for weight loss as well as signs of rectal bleeding and diarrhea. The disease activity index (DAI) was determined according to the parameters outlined in Table 4^34^. On day 10 (d10) of DSS administration, Heparin anti-coagulated blood samples were collected by retro-orbital bleeding. MINDRAY BC-3000 PLUS Automatic Hematology Analyzer was used to test hematological parameters. In addition, mice were sacrificed and tissue segment were taken from the distal, proximal colon and cecum of each mouse for histological assessment. Histological evaluation of DSS-induced colitis severity was performed as follows, the colons were stained with Hematoxylin-Eosin and evaluated by the blinded pathologist under a light microscope. All the colons were scored as Table 2 described according to the reference report^35^. For each animal, two sections approximately 400 μm apart were scored and averaged.

### Statistical analysis

All data are means of at least 3 independent experiments and presented as mean ± SD (standard deviation) or mean ± SEM (standard error of the mean). The data were subjected to analysis of variance (ANOVA). One, two and three asterisks represented a significant difference two treatment groups with *P* < 0.05, *P* < 0.01 and *P* < 0.001, respectively.

## Author Contributions

L.L., D.G. and L.W. conceived and designed the experiments. L.H., Y.W. and K.L. collected berry extracts. Z.W. and L.Y. performed the cell culture and most of experiments. L.L., L.W., and D.G. supervised data collection, performed the analysis and wrote the manuscript. X.Z. analyzed the data. L.L., L.W. and Z.W. contributed equally to the study. All authors discussed the results and commented on the manuscript.

## Supplementary Material

Supplementary Information Supplementary information

## Figures and Tables

**Table 1:** Anthocyanin-rich fractions (ARFs) from crude extracts (500 mg)

Fruit	ARFs (mg)
Red Raspberry (RR)	86.7 ± 0.43
Mulberry (MB)	92.4 ± 0.08
Black Raspberry (BR)	93.1 ± 0.08
Triple Crown (TC)	77.0 ± 0.10
Shawnee (SH)	58.8 ± 0.19
Chester (CH)	59.2 ± 0.14
Kiown (KI)	58.2 ± 0.25

The content of ARFs is expressed as mean ± SD.

**Table 2:** Scoring system for inflammation-associated histological changes in the colon

Score	Histologic changes
0	No evidence of inflammation
1	Low level of inflammation with scattered infiltrating mononuclear cells (1–2 foci)
2	Moderate inflammation with multiple foci
3	High level of inflammation with increased vascular density
4	Maximal severity of inflammation and loss of goblet cells

**Table 3:** Effect of RR-ARFs on damage score

Group	Damage score
Normal	0
2% DSS	2.15 ± 0.30###
2% DSS + 20 mg/Kg RR-ARFs	1.15 ± 0.25**

**P < 0.01 vs the 2% DSS model,

^###^*P* < 0.001 vs the normal group. Damage score is expressed as mean ± SEM.

**Table 4:** Scoring system to calculate the disease activity index (DAI)

Score	Weight loss	Stool Consistency	Blood in feces
0	None	Normal	None
1	1–5%		
2	6–10%	Loose Stool	Occult bleeding
3	11–20%		
4	>20%	Diarrhea	Gross bleeding

The DAI value is calculated as the scores of weight loss, stool consistency, and blood feces divided by 3.Weight loss is always calculated to day 1 of each group.
