# Supplementary material for: Anthocyanin-rich fractions from red raspberries attenuate inflammation in both RAW264.7 macrophages and a mouse model of colitis
Source: Sci Rep. 2014 Aug 29;4:6234. doi: 10.1038/srep06234 (PMC4148654; doi:10.1038/srep06234)
Supplement: Supplementary Information [file srep06234-s1.doc]

**Supplementary information**

**Anthocyanin-rich fractions from red raspberries attenuate inflammation in both RAW264.7 macrophages and a mouse model of colitis**

Li Li^1^*,Liyan Wang^2^*, Zhiqin Wu^1^*, Lijun Yao^1^, Yonghou Wu^3^, Lian Huang^1^, Kan Liu^4^, Xiang Zhou^2^ & Deming Gou^1,2^

^1^ College of Life Sciences, Shenzhen Key Laboratory of Microbial Genetic Engineering, Shenzhen University, Shenzhen 518060, China;

^2^ College of Life Sciences, Department of Marine Science and Bio-Pharm, Shenzhen Key Laboratory of Marine Bioresourse and Eco-environmental Science, Shenzhen 518060, China;

^3^ College of Animal Science and Technology, Northwest A&F University, Yangling, Shaanxi 712100, China;

^4^ College of Life Sciences, Shenzhen key laboratory of synthetic biology, Shenzhen University, Shenzhen 518060, China.

*These authors contributed equally to this work.

**Corresponding Authors**

E-mail: (D.G.) dmgou@szu.edu.cn. Fax: 86-755-26534274. Tel.: 86-755-26557690


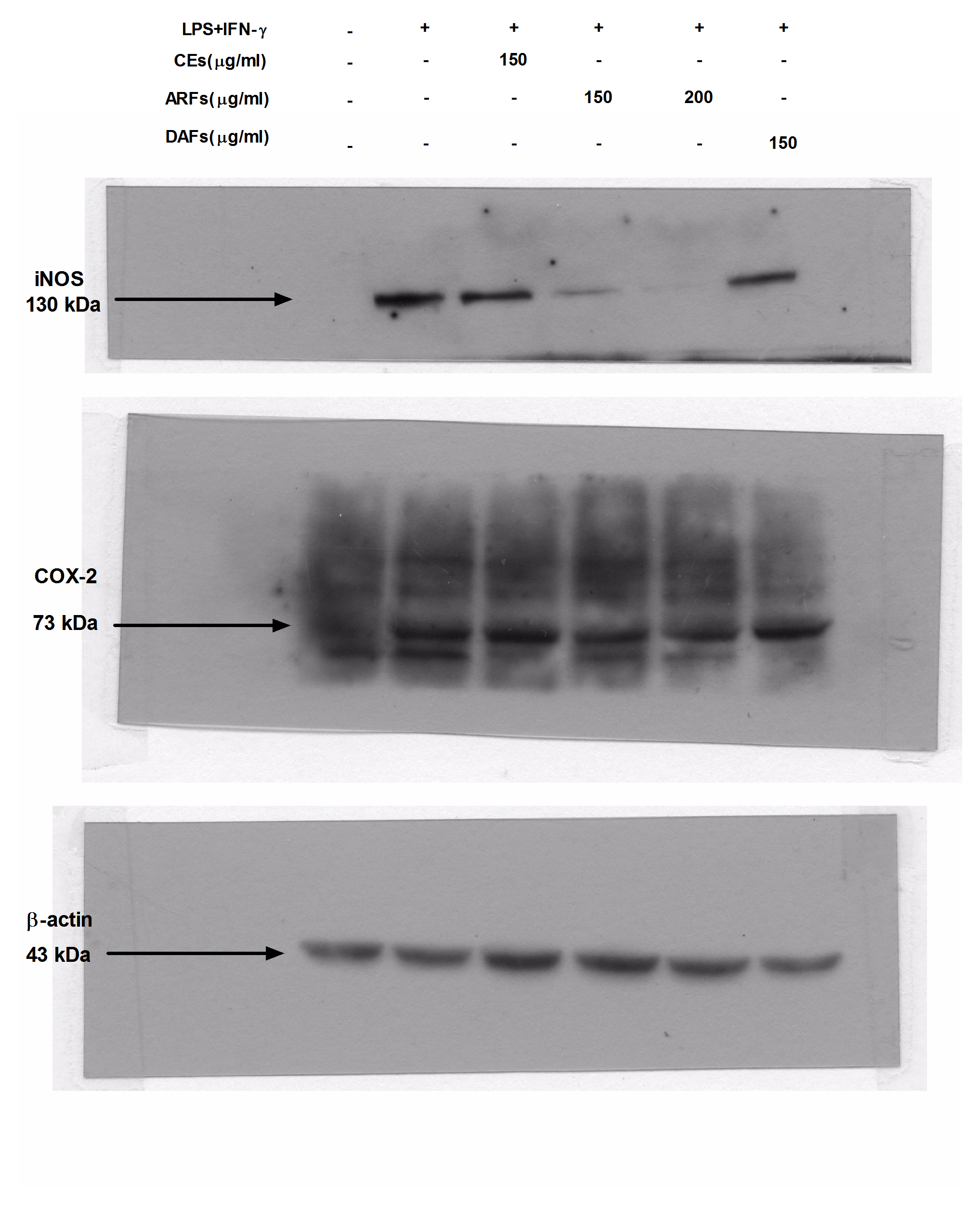


**Figure S1.** Full-length blots of iNOS, COX-2 and β-actin.

Regions of interest are highlighted in Fig. 3e.


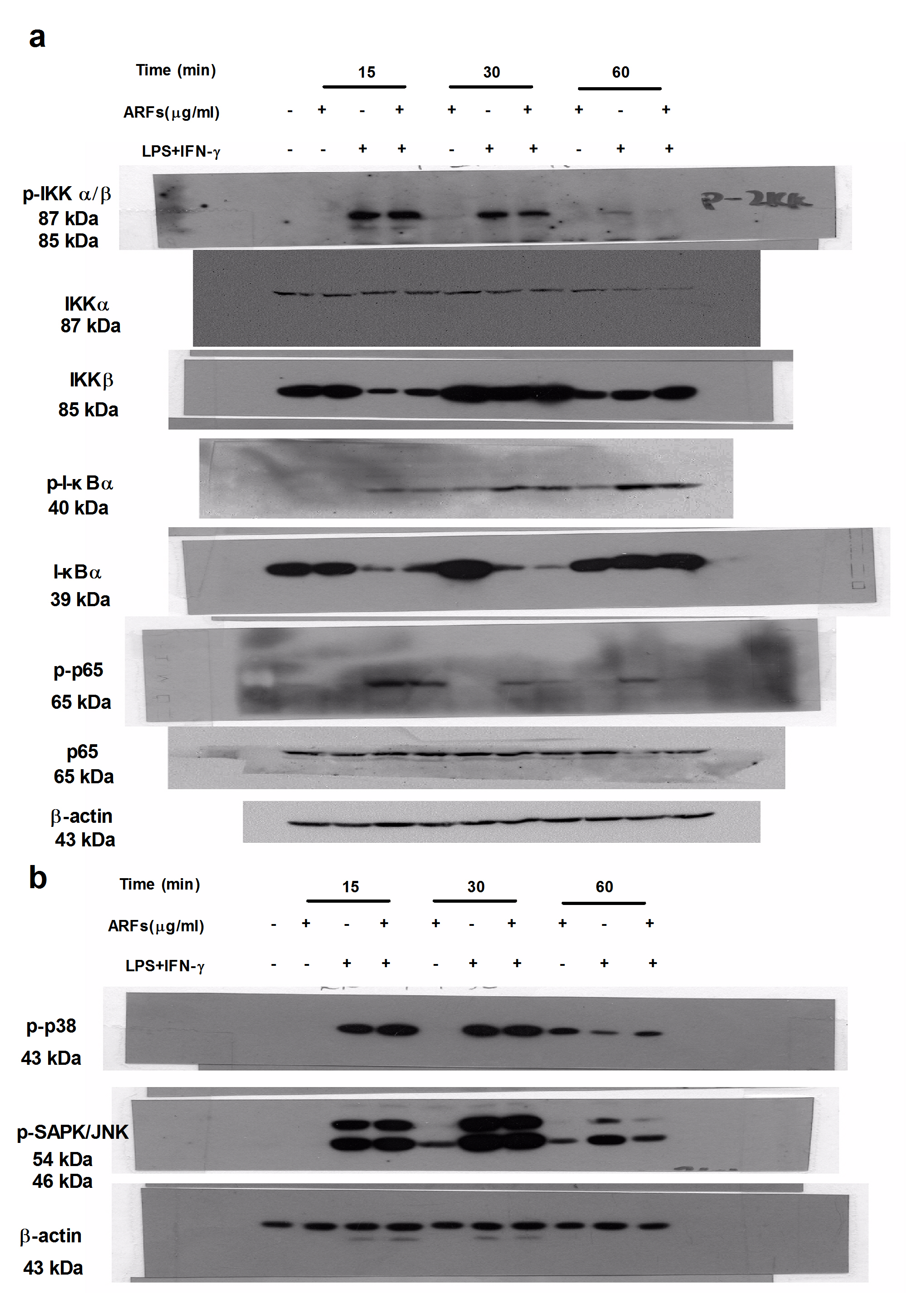


**Figure S2.** Full-length blots of IKK and MAPK signaling pathway.

Regions of interest are highlighted in Fig. 6a and 6b.
